# A Study on Prevalence of Chewing Form of Tobacco and Existing Quitting Patterns in Urban Population of Jamnagar, Gujarat

**DOI:** 10.4103/0970-0218.62560

**Published:** 2010-01

**Authors:** Urvish Joshi, Bhavesh Modi, Sudha Yadav

**Affiliations:** Department of Community Medicine, Smt NHL Municipal Medical College, Ahmedabad - 380 006, India; 1 Department of Community Medicine, PDU Medical College, Rajkot, India; 2 Department of Community Medicine, Shri MP Shah Medical College, Jamnagar - 361 008, India

**Keywords:** Family exposure, prevalence, tobacco chewing, tobacco hazards, quitting

## Abstract

**Background:**

Awareness towards tobacco hazards has increased with time but its role alone towards cessation is questionable. With widespread menace of tobacco in developing countries like India, not much tobacco chewing prevalence and their quitting patterns data are available in urban Saurashtra region.

**Objectives:**

1. To find out prevalence of various forms of chewing tobacco and quitting attitudes in urban Jamnagar. 2. To study quitting patterns in relation with age of habit initiation, family background and habit duration.

**Materials and Methods:**

It was a cross-sectional study involving 2513 individuals as study population by 30-cluster sampling method. The study was carried out between June 2007 and March 2008. Pre-set, pre-tested questionnaire was used for interview purpose and the statistical analysis was done on proportion basis.

**Results:**

About 37.2% of study population was ever-tobacco-chewers; 32.9% of them were current-chewers and 4.3% were quitters. Approximately 28.4% of current-consumers were willing to quit. Mawa-masala (63.7%) and Gutka (57.6%) were preferred forms of chewing tobacco and 57.5% of the current-chewers chewed tobacco six to eight times a day. Tobacco initiation age between 20 and 30 years was commoner among quitters (84.2%), while a little younger in current-consumers (76.5%). About 58.3% quitters and 74.0% chewers showing willingness to quit had not consumed tobacco for more than five years, 63.8% of current-chewers had a family member consuming tobacco. With initiation of health problems, 72.2% subjects quit and 55.5% of them already knew about health hazards.

**Conclusions:**

Every 4 out of 10 residents was found to be exposed to chewing tobacco. With Mawa-masala and Gutka being the predominant forms, habit onset in late adolescence, years of consumption and family exposure seem to be hampering quitting. Awareness about tobacco hazards alone does not appear to be resulting in successful quitting.

## Introduction

Nearly two-thirds of world's smokers live in just ten countries and more than 40% live in just two countries i.e. China and India. India bears around 10% share of total smokers in the world.(1)

According to NFHS-III, in India, 55.8% male, 10.8% female in the age group of 12 to-60 years have been found to be consuming tobacco. Among males, 32.7% smokers while 36.5% tobacco chewers are reported, while among females; it is reported to be 1.4 and 8.4%, respectively.(2)

Tobacco has always been a menace in developing countries like India and chewing tobacco apparently is widely prevalent in the state of Gujarat, unfortunately, not much data are available pertaining to prevalence and quitting patterns for chewing form of tobacco in urban regions of Saurashtra especially Jamnagar, which is a rapidly developing industrial and petroleum hub in western part of India.

Pan masala is a mixture of nuts, seeds, herbs and spices, which is served after meals in India. In the aforesaid region of Gujarat, where the study is carried out, it is customary to add tobacco in pan masala and hence it is a common form of tobacco chewing. Another form, Gutka, is a preparation of crushed betel nut, tobacco, catechu, lime and sweet or savory flavorings.

Awareness towards hazardous health effects of tobacco has increased with time but its role alone towards attainment of tobacco cessation is questionable.

As per India's Cigarette and Other Tobacco Product Act 2003 (COTPA), selling tobacco to minors or selling of tobacco by minors (under the age of 18) is legally forbidden and violation of the same is a punishable offence. Same applies to selling of tobacco containing items within 100 yards radius of any educational premise.

From 31^st^ May, 2009, as per the amendment in COTPA 2003, the pictorial as well as text warning covering at least 40% of the total area of advertisement is mandatory in India.

For effective formulation of quitting strategies, knowledge regarding quitting behavior is necessary to study and hence a cross-sectional study was carried out in Jamnagar city to find out the prevalence of chewing tobacco along with prevalence of quitters, their reasons for quitting, quitting behavior in existing consumers and associated factors.

## Materials and Methods

It was a cross-sectional study in which based on the results of prior pilot study on prevalence of tobacco use in urban Jamnagar, 2513 individuals were selected by 30-cluster sampling method from total 20 different wards of Jamnagar city.

The study was carried out between June 2007 and March 2008. Pre-set, pre-tested questionnaire was used for interview purpose and the statistical analysis was done on proportional percentage basis, study variables being Tobacco chewing, chewing forms, quitting, quitting reasons and health hazards, age of tobacco initiation, duration of tobacco chewing, daily frequency, family exposure of tobacco and knowledge regarding health hazards of tobacco.

Following study terms were taken into consideration: Current-Tobacco-Chewers were those who had chewed tobacco regularly and chewed at least once on an average each day, during the previous 30 days at the time of study. Tobacco-Chewing-Quitters were those who chewed tobacco in the past but had quit and not chewing presently. The current-tobacco-chewers and past-tobacco-chewers together formed the term Ever-Tobacco-Chewers. Never-Tobacco-Chewers were the persons who never chewed tobacco in their lives.

The recorded reasons for quitting were inclusive of psychological i.e. pressure from spouse and/or family, preaching by religious leaders and taking subsequent vows of quitting tobacco, advice by doctors on health grounds, advice by peers/friends/relatives on social grounds and physical i.e. initiation of health problems such as respiratory problems like coughing, breathlessness, short breathing and wheezing; reduced widening of mouth, weight loss, appearance of pre-cancerous lesions like leukoplakia and decreased working capacity.

## Results

About 37.2% of the total study population was found to be exposed to tobacco chewing either in past or present, Out of which, 32.9% were current-chewers and 4.3% had already quit their habit. A total of 186 out of total 2513 subjects (7.4%) were found to be tobacco smokers, while 827 were reported to be tobacco chewers (32.9%). 103 subjects (about 4%) were found to be consuming both smoking and smokeless forms of tobacco.

Mawa-masala was the preferred form of chewing found in 63.72% chewers followed by Gutka in 57.6%. Approximately 24.5% of current-consumers used tobacco in pan, while 48 (5.8%) used bajar. Mean age of tobacco initiation was found to be 26.5 years in case of smokers, while it was 23.6 years in case of tobacco chewers.

Among all study subjects, smoking prevalence was found to be 0.4% in late adolescence, which rose sharply to 8.11% in the age group of 17 to 19 years and was found highest between 25 and 35 years (21.36%).

**Figure 1:**
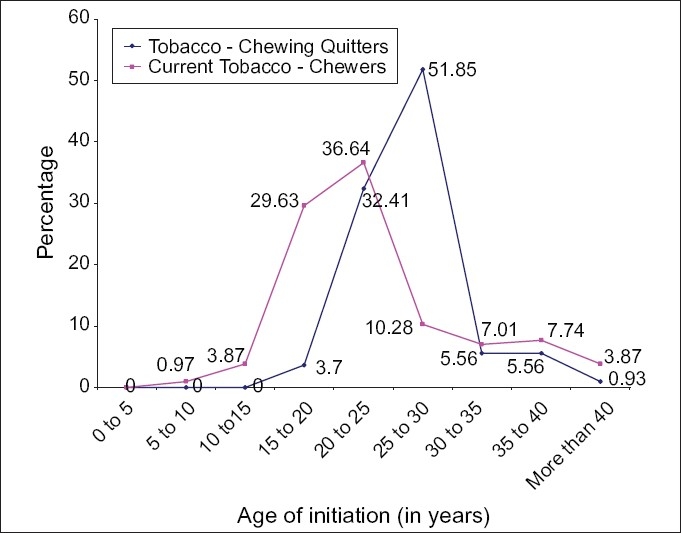
Age of initiation of tobacco in current-tobacco-chewers and quitters

Tobacco chewing prevalence was almost 10% in the age group of 13 to 17 years, which rose to 51.3% among 17 to 19 years. It was found highest in 45-55 years of age group (76.1%), followed by 56.6% in 35 to 45 years of age. About 28.4% of the current-consumers showed willingness to quit their habit. Majority of quitters (84.2%) had started tobacco between the age of 20 and 30, while the year span was five years earlier i.e. initiation between 15-30 years in 76.6% of current-tobacco-chewers. Almost 30% of current-consumers had started before they completed 20 years[Figure 1].

Approximately 58.3% of quitters and 74.1% of current-chewers who showed willingness to quit had not consumed tobacco for more than five years. Among those who were not willing to quit tobacco, 80% had chewed tobacco for more than five years.

About 63.9% of current-tobacco-chewers had a family member consuming tobacco in any form, while 48.2% of quitters had a history of any family member consuming tobacco in any form. Among the successful quitters, major reasons for quitting were found to be initiation of health problems (72.2%), which included respiratory problems like coughing, breathlessness, short breathing and wheezing; reduced widening of mouth, weight loss and decreased working capacity cumulatively. Vows which were self-offered or resulted from positive preaching from the local religious leaders towards quitting of tobacco played a role in 60.2% of cases, while the reason was familial pressure either by spouse or by other family member(s) in 40.7% of the cases.

Approximately 19.4% of quitters did not have a specific reason to quit, while among the current-tobacco-chewers, who were willing to quit, had social pressure (48.5%) and religious vows (42.1%) as the major reasons behind their willingness to quit.

Out of those who quit after initiation of health problems, 55.6% already knew about health hazards of tobacco but did not bother until any health disturbance occurred to them.

## Discussion

The study results suggest a wide prevalence of tobacco chewing in the study population. Almost one-fourth of the residents were found to be exposed towards the habit of chewing tobacco. More than 30% of the individuals were chewing tobacco at the time of study, which concurs with Sen *et al*. (2000) who found the prevalence rate of tobacco chewing to be 36% in males and 19% in females.(3)

In the present study, the mean age of tobacco initiation was found to be 26.5 years in case of smokers, while it was 23.6 years in case of tobacco chewers. No national level data were available for the same but several studies by Kapoor *et al*. (1995),(4) Babu *et al*. (1978),(5) Sen *et al*. (2002),(6) Singhi *et al*. (1987)(7) etc. accord with the findings of early age of initiation of the habit.

In GYTS,(8) among 13 to 15 years, 25.1% subjects were found to be ever-tobacco-users, 17.5% subjects were current-tobacco-users, which comprised of 17.6% smokeless form of current tobacco use and 8.3% of smoking form of tobacco use while in current study, smoking prevalence was almost nil among 13 to 15 years but significant in late adolescence (8.11%) and was highest between 25 to 35 years (21.36%). Similarly, tobacco chewing prevalence was almost 10% in the age group of 13-17 years, while more than half of the subjects belonging to the age group of 17 to 19 years (51.3%) were found chewing tobacco. It was highest in 45-55 years of age group (76.1%), followed by 56.6% in 35 to 45 years of age. Sen U and Basu A (2000) reported that increased tobacco use was associated with older age groups.(3)

Only 4.3% individuals were found to have quit tobacco successfully. Anantha *et al*. (1995) in their interventional study found the quitting rate to be 30.2% among male tobacco chewers.(9)

Sargent *et al*. (1998)(10) reported in their study that 25.7% subjects wanted to quit smoking now, which was almost similar to the observations in the present study for willingness to quit (28.42%).

Mawa-masala and Gutka were the predominant forms of chewing tobacco, prevalence being much higher than what Gupta *et al*. (1996) found about betel quid with tobacco (27.1%).(11)

More than half of the chewers were chewing tobacco as many as six to eight times a day. Habit initiation in late adolescence may contribute towards strengthening the addiction, which eventually could hamper quitting efforts. In the present study, majority of quitters were found to have initiated their habit after age 20. More than three-fourth of current-chewers had started chewing before the age of 20, which supports results of Sen *et al*. (2000) (one-third of men starting tobacco before 20) and Nisar *et al*. (2007) (90% of study subjects having started consuming tobacco before 20 years of age).(3,12)

More than half of quitters (58.33%) and almost three-fourth (74.04%) of current-chewers who showed willingness to quit had not had the habit lasted for more than five years. Eighty percent of those with reluctance towards quitting had chewed tobacco for more than five years.

More than half of current-tobacco-chewers had a family member consuming tobacco in any form positioning second to Babu *et al*. (1978) report, which shows positive association of tobacco smoking with presence of smokers in the family in urban areas of Delhi.(5)

Health problems were the major reasons behind quitting, which concur with results of Sharma *et al*. (1990) and Hymowitz *et al*. (1997).(13,14) Other major reasons were religious vows and social pressure.

Harmful effects of tobacco were found to be widely known concurring the finding of Basu *et al*. (1992) that three out of four persons knew the problems of smoking.(15) More than half of those who quit chewing tobacco after appearance of unfavorable health events knew earlier about the harmful effects of tobacco but did not pay much attention till it started showing.

People know much about the health hazards of tobacco and that merely is not sufficient to stop them from taking up or from continuing the habit. There is a need to develop multifactorial tobacco quitting strategies focusing on early age intervention and covering the addict along with his surrounding environment.
